# Patient's Preference on Neurosurgeon's Attire and Appearance: A Single Center Study in Korea Cross-Sectional Study

**DOI:** 10.1155/2019/3893049

**Published:** 2019-04-09

**Authors:** Hyun Woong Mun, Ji Hee Kim, Jun Hyong Ahn, In Bok Chang, Joon Ho Song, Jae Keun Oh

**Affiliations:** Department of Neurosurgery, Hallym University Sacred Heart Hospital, Anyang, Republic of Korea

## Abstract

**Objective:**

This study was performed to assess neurosurgery patients' preference for surgeon's attire and appearance in the hospital.

**Methods:**

A total of 100 patients were investigated using a questionnaire comprising 13 questions. We first asked patients about neurosurgeon's appearance including accessories, hair color, mustache, and beard. Then, based on their preferences, they were asked to rank a series of photographs which illustrated a variety of neurosurgeon attires worn by a doctor.

**Results:**

Professional attire with white coat was the most ideal for patients compared to any other attire (preference scale 5.28 ± 1.24), and there was a significant preference gap between wearing a white coat and not wearing a white coat (p <0.01). Patients expressed a preference for neurosurgeon's shoes (30%) but the majority of the respondents answered that it does not matter which shoes their neurosurgeon wears (43%). Furthermore, the patients did not have any preferences regarding accessories, dyeing, and mustache or beard (58%, 67%, and 51%, respectively), and they did not have negative view towards doctor wearing accessories (71.7%) and growing mustache or beard (71.6%).

**Conclusion:**

The patients in this study preferred professional attire along with a white coat compared to any other form of outfit. This result is similar to those of many other studies conducted in other departments or other countries. However, patients did not have a strong negative view on accessories, dyeing, and facial hair. With regard to medical training, patients did not show a preference for their neurosurgeon's educational background.

## 1. Introduction

Originally, the importance of doctor's appearance in the patient-physician relationship was emphasized by Hippocrates, who stated that doctors “must be clean in person, well dressed, and anointed with sweet-smelling unguents” [1]. The white coat and formal appearance symbolize medical professionalism and have been worn by all doctors since the 19th century. Studies have demonstrated that personal attributes of doctors, such as clothing and cleanliness, play a crucial role in the development of patient-physician relationship [2].

In the recent years, use of white coat has somewhat declined mainly due to concerns over the transmission of hospital acquired infections. The Department of Health in UK published guidelines in 2007 that it is better for doctors to remove white coats, jackets, and ties and roll up their shirt sleeves (bare below the elbows, BBE) [3]. However, other studies have stated that compared to other formal attires, the ideal attire according to the BBE regulation is not well accepted by the public [4]. Thus, it might be somewhat difficult to establish a patient-doctor relationship when doctors wear the BBE attire.

Similarly, other departments, such as internal medicine, orthopedics, and ophthalmology, as well as many countries, such as England, USA, and other western countries, have shown interest in the establishment of patient-doctor relationships by taking doctor's dress code into account. However, the importance of the doctor's appearance in relation to other professional attributes is largely unknown in Korean neurosurgery departments.

The purpose of our study was to determine the preference for doctor's appearance and attire by investigating the opinions of neurosurgery patients and their relatives. Moreover, the uniqueness of this study is that we also investigated their preferences with respect to age and other factors when choosing their doctor in charge, while taking into consideration the importance of educational background in Korea.

## 2. Materials and Methods

The study consisted of a short questionnaire (Table 1), which was administered to patients visiting the neurosurgery outpatient department or for hospitalization at Hallym Sacred Hospital. The survey content was designed considering patients' preferences and the Korean cultural context and was agreed upon by all participants prior to collecting their data [5].

The patients and visitors were aged over 20 years and were selected randomly over several days from January to February 2018. Those with cognitive or visual impairment were excluded.

**Figure 1:**
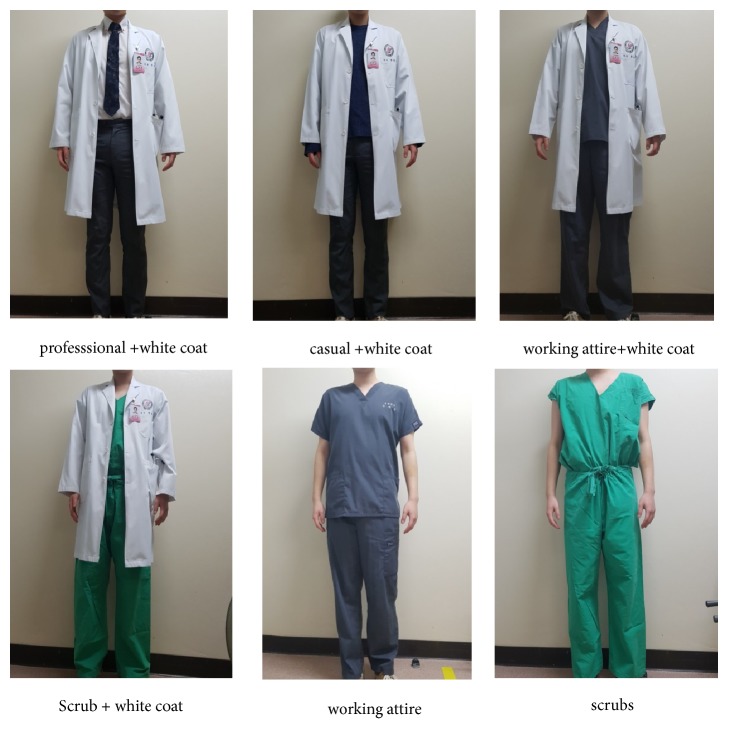
A doctor dressed in six different styles of clothing.

The survey questionnaire (Table 1) consisted of ten questions, which were divided into three sections. In the first section (Q1-Q6), the respondents were asked about their view on neurosurgeon's specific appearances, which was answered as “prefer” or “no preferences.” In the second section (Q7-Q8), respondents were asked about their preferred doctors' traits when choosing the neurosurgeons. Finally in (Q10), respondents were shown clinical photographs of a doctor dressed in six different styles of clothing (Figure 1). They were asked to rank the six styles based on their preferences (from 6-most preferred to 1-least preferred) [6].

A total of 100 patients (47 males and 53 females) participated in this cross-sectional survey which assessed the desirable and undesirable traits of their neurosurgeon according to their preferences. Patients' mean age range was from 40 to 49, and all of them were Koreans [7].

SPSS version 18.0 was used for statistical analyses. Chi-square analysis was performed to determine if a correlation exists between patients' demographic characteristics (gender and age) and patient preferences for neurosurgeon appearance. The ranking of the six different clothing styles was presented as means and standard deviations (SD). White coat preferences were analyzed by the mean values between the white coat (1, 2, 3, and 4) group and the nonwhite coat (5 and 6) group using paired T-test.

The analysis of one way variance (ANOVA) was performed to investigate the correlation between patients' demographic characteristics (gender and age) and white coat preferences [8].

## 3. Results

The patients exhibited varied preferences regarding the neurosurgeon's appearances (Table 2). Patients expressed a preference for neurosurgeon's dress shoes (30%) but the majority of the respondents answered that the type of shoe worn by the neurosurgeon does not matter (42%). There was no significant correlation between patient demographics (gender and age) and preference for shoes (p=0.735, p=0.512).

Likewise, the patients favored more the surgeons having their nametag on (76%), and females preferred nametags than men did (86.8%, p=0.01). Furthermore, patients did not have any preferences regarding wearing accessories, dyeing, and having a mustache or beard (58%, 67%, and 51%, respectively), and there was no negative view towards accessories (71.7%) and mustache or beard (71.6%). With regard to dyeing the hair, male patients tended to have a more negative view than women did (53.2%, p<0.01).

Most respondents considered the ideal age of the neurosurgeon in charge to be 50-59 years (31%), and “no preference” item was the similar percentage (29%). By the age stratification analysis, those under 49 years of age favored neurosurgeons aged 40-49 years, while those above 49 years of age preferred neurosurgeons aged 50-59 years (p<0.01).

People were in favor of short hair on male physicians (49%); however, there were a slightly higher number of respondents who did not have any preference (51%). With respect to respondents' age, older respondents preferred short hair (p=0.015).

In addition, most people chose the physician based on other's recommendation (31%) followed by the center where the MD worked (28%). Patients considered that professors should have the most formal appearance (69.8%), followed by nurses (20.8%) (p=0.159). Patients considered that professors (69%) should be the tidiest, and other factors showed no effect on patient preferences.

In the perspective of attire, professional attire along with white coat was considered the most ideal for patients than any other attire (Table 3, p<0.01). This was followed by casual attire and white coat, and there was a statistically significant gap between scrubs. Furthermore, there was a statistically significant difference in the preference for neurosurgeons wearing a white coat (4.29, 1.93; p<0.01).

However, the demographic factors (gender and age) had no significant effect on patients' preference for a white coat (p=0.198, p=0.528).

## 4. Discussion

Regarding attire, there was a significant preference for the traditional white coat and professional attire. This finding is similar to those of many other studies conducted worldwide in a variety of settings, although we cannot compare these studies to the Korean context because there have been no studies in the Korean neurosurgery or any other department in Korea.

There was a study on patients' attitudes toward surgeons' attire in an outpatient clinic setting. The patients did not prefer surgeons wearing white lab coats but preferred those wearing surgical scrubs; however, this finding was not statistically significant [9].

The difference in the outcomes of our study and that of Edwards et al.'s study might be due to the contents of the questionnaire. The latter inquired about the responsibility of the surgeon to wear a white coat, while our study classified the attire according to patients' preferences.

In a study by Rehman et al., similar to the findings of the present study, the respondents overwhelmingly favored professional attire with white coats for physicians (76.3%, p=0.0001), which asserted that wearing a professional attire during consultation with a patient may help build trust and confidence [10].

Regarding physical appearance, there was no significant preference for neurosurgeons in terms of piercing, facial hair, and hair length. The preference for having a nametag on (76%) might be to identify the doctor by their name. With regard to accessories, such as necklace, wristlet, and rings, most of the patients did not have a negative view of them. Previous studies have shown that piercings adversely affects the level of patient's confidence. Johnson et al. reported that a visible piercing on a medical care provider directly affects patients' perception of and trust in the provider's capabilities [11]. The discrepancy between the findings of Johnson et al.'s study and our study may be explained by doctors in Korea having a tattoo or wearing obtrusive accessories being few in number, and the questionnaire used in this study only included items, such as necklace, wristlet, and rings.

However, the preferences were diverse according to the departments. In Kanzler et al.'s study in a dermatology setting, the patients preferred dermatologists to wear a nametag, white coat, avoid open shirts, earrings, and long hair [12]. In a study in an ophthalmology department, patients preferred the ophthalmologists to wear the white coat and nametag but were tolerant on shoes, hair length, and facial hair and had a negative view on tattoos or piercings [13]. Our study suggests that in the neurosurgery setting, patients were more focused on the tidiness of the attire and the quality of their neurosurgery care as was seen in patients choosing the neurosurgeon based on the formal attire rather than personal traits.

With regard to the criteria for choosing a neurosurgeon, others' recommendation was the most important (31%), and being a university graduate and center-trained MD were not important factors compared to others' recommendation or the center where the MD worked (13% vs. 59%). Moreover, female patients were less likely to be interested in the educational background of the doctor (p<0.01). In a study in the orthopedic department, Abghari et al. reported that patients' concerns about their orthopedic surgeon seemed to not be based on the quality of education and experience of their physician [14]. Our patients also had a similar tendency when selecting the surgeon based not on the surgeon's educational background but on his or the patient' current circumstances.

Our study also examined patients' preferences for the doctor's appearance with respect to the demographics (gender and age). Female patients tended to prefer doctors having a nametag on; they did not prefer dyeing and choosing neurosurgeon based on others' recommendation. Regarding the preferences for attire or white coat, there were no significant differences between the male and female patients but female patients had a stronger dislike for a working attire without a white coat (p=0.01). Overall, female patients favored formal appearance more except for dyeing despite other provisions being statistically not significant.

With respect to patients' age, aged patients had a preference for short hair for male neurosurgeons, and the patients considered the ideal age of their doctor in charge as the same age bracket as theirs. Patients below 50 years of age preferred neurosurgeons aged 40-49 years, while those over 50 years of age preferred neurosurgeons aged 50-59 years (p<0.01). Ardolino et al. found that young respondents (those below 55 years) preferred surgical scrubs while those aged 55 years or older preferred a short-sleeved shirt [15]. Lill and Wilkinson also reported in their study that in terms of preferences by age, older patients preferred a more formal attire than did their younger counterparts [16]. However, we found no such association between the age of participants and their responses to questions in our study, with the exception of a weak correlation between advancing age and a preference for the white coat. In contrast to what we expected to find, older individuals who responded to our survey did not place greater emphasis on appearance and formal attire. We found formal attire and the white coat to be universally accepted and the highest-ranked choice irrespective of patient's age or gender.

Patients' preferences for doctors in terms of style have been investigated in numerous contexts, and we compared the other papers' consequences. However, it is appropriate considering the cultural differences in the settings. Many western countries, such as USA [10], England [17], and Brazil [18], are in favor of the white coat and prefer a formal attire. In Japan, a study investigated patients' preferences for doctors' attire, which also found a preference for a white coat and formal attire. Furthermore, this study revealed that wearing a white coat could favorably influence patients' confidence in the patient-doctor relationship in all types of practice [19]. Likewise, several studies have shown that regardless of the skin color, most patients favored the white coat and formal attire.

It has been known that a doctor's attire may be important in the success of the patient-doctor relationship [20]. The present study supports this finding. On September 2007, the British Department of Health launched the BBE dress code to control the infection rates among hospital staff, which mandates that the staff should wear short sleeves and should not wear ties, wristwatches, or jewelry when carrying out clinical duties, but there was a lack of discussion regarding the professionalism of doctor when this policy was performed. However, many other studies have argued that work attire with respect to professionalism and cleanliness may challenge the patient-doctor relationship; many patients regard the long-sleeved shirt and tie as the most professional attire. There was no significant gap between working attire and professional attire in terms of risk of transmitting hospital acquired infection; thus they regarded long-sleeved shirt and tie as the ideal attire for a doctor [4]. Likewise, the patients in our study preferred formal attire with white coat; they did not have any negative view of doctors wearing accessories. To build a stronger patient-doctor relationship, it is reasonable for a neurosurgeon to wear a white coat with formal attire.

This is the first study to evaluate the association between neurosurgeons' attributes and patient preference in Korean neurosurgery. Our patients preferred professional attire along with white coat than any other type of attire. These results are similar to those of many other studies conducted in other departments and other countries. We thought that this was on account of personal attributes of doctors, such as white coat, clothing, and cleanliness, playing a crucial role in the development of patient-physician relationship. However, in the present study, patients did not have a strong negative view on accessories, dyeing, and facial hair. With regard to medical training, patients do not show a preference for where their neurosurgeon's educational background.

There is also a trend of banning white coats and formal attire in order to prevent hospital-acquired infection by the Korea Ministry of Health and Welfare. It may sound reasonable if doctors wear short-sleeved clothing when delivering patient care and remove accessories, so healthcare associated infection through doctors will be lower. However, many studies proved that this hypothesis lacks evidence. For example, national evidence-based guidelines in NHS hospitals in England 2014 demonstrated class D evidence with regard to wearing short-sleeved clothing and not wearing white coats [21]. Complying with groundless trend may disturb building a firm relationship between the neurosurgeon and patients; thus, we are better off wearing a white coat along with formal attire.

## Figures and Tables

**Table 1:** The questionnaire.

Questions

what is your gender?
(1) male (2) female
what is your age?
(1) under 30 (2) 30-39 (3) 40-49 (4) 50-59 (5) over 60
(1) What shoes do you prefer your neurosurgeon to wear?
(1) Dress shoes (2) Sneakers (3) Clogs (4) sandals (5) No preference
(2) Do you prefer a nametag?
(1) prefer (2) No preference
(3) Do you have a negative view on wearing accessories (necklace, wristlet, and ring)?
(1) Yes (2) No preference
(4) Do you have a negative view on dyeing neurosurgeon's hair color?
(1) Yes (2) No preference
(5) Do you have a negative view on your neurosurgeon's mustache or beard?
(1) Yes (2) No preference
(6) Do you have a negative view on long hair? (in case of male doctor)
(1) Short hair(showing his ear) (2) long hair (disclosing his ear ) (3) No preference
(7) Do you have a preferred age range?
(1) 30-39 (2) 40-49 (3) 50-59 (4) 60-69 (5) No preference
(8) Which criteria do you take when choosing your neurosurgeon?
(1) Graduated university (2) the center MD working (3) the center MD trained (4) Other's recommendation (5) No preference
(9) Do you think who should be neat most?
(1) Professor (2) residents (3) Intern (4) nurse

**Table 2:** Responses to survey questions.

	Dress shoes	Sneakers	Clogs	sandals	No preference
Q-1. (prefer shoes)	30 (30%)	16 (16%)	8 (8%)	3 (3%)	43 (43%)

	Yes	No preference			

Q-2. (prefer nametag)	76 (76%)	24 (24%)			
Q-3. (negative view on accessory)	42 (42%)	58 (58%)			
Q-4. (negative view on dyeing)	33 (33%)	67 (67%)			
Q-5. (negative view on mustache or beard)	49 (49%)	51 (51%)			

	Short hair	Long hair	No preference		

Q-6. (prefer hair length in male)	49 (49%)	0 (0%)	51 (51%)		

	30-39	40-49	50-59	60-69	No preference

Q-7. (preferred age)	7 (7%)	27 (27%)	31 (31%)	6 (6%)	29 (29%)

	Graduated university	the center MD working	the center MD trained	Other's recommendation	No preference

Q-8. (preferred criteria When to choose neurosurgeon)	11 (11%)	28 (28%)	2 (2%)	31 (31%)	28 (28%)

	Professor	Residents	Intern	nurse	

Q-9. (the most tidy occupation)	69 (69%)	11 (11%)	2 (2%)	18 (18%)	

**Table 3:** Comparison with white coat and non-white coat group.

	White coat		Not wearing white coat			p-value
Total preference scale	4.29 (±0.39)		1.93 (±0.78)			<0.01

	Man		Woman			p-value

White coat	4.23 (±0.47)		4.33 (±0.30)			0.198
Not wearing white coat	2.03 (±0.93)		1.84 (±0.60)			0.219

	<30	30-39	40-49	50-59	>60	p-value

White coat	4.38 (±0.27)	4.28 (±0.30)	4.36 (±0.28)	4.19 (±0.55)	4.24 (±0.45)	0.528
Not wearing white coat	1.75 (±0.55)	1.94 (±0.60)	1.79 (±0.57)	2.11 (±1.09)	2.05 (±0.89)	0.498

## Data Availability

The [SPSS] data used to support the findings of this study have been deposited in the [Figshare] repository (DOI: 10.6084/m9.figshare.7399193), are included within the article and within the supplementary information file(s), and were supplied by [Figshare] under license and so cannot be made freely available.

## References

[1] Potter P. (1984). *Hippocrates*.

[2] Van Dulmen A. M., Verhaak P. F. M., Bilo H. J. G. (1997). Shifts in doctor-patient communication during a series of outpatient consultations in non-insulin-dependent diabetes mellitus. *Patient Education and Counseling*.

[3] Jacob G. (2007). *Uniforms and Workwear: An Evidence Base for Developing Local Policy*.

[4] Baxter J., Dale O., Morritt A., Pollock J. (2010). Bare below the elbows: professionalism vs infection risk. *The Bulletin of the Royal College of Surgeons of England*.

[5] Lee C., Chung C. K., Kim C. H., Kwon J. (2018). Health care burden of spinal diseases in the republic of korea: analysis of a nationwide database from 2012 through 2016. *Neurospine*.

[6] Eguchi Y., Suzuki M., Yamanaka H. (2017). Assessment of clinical symptoms in lumbar foraminal stenosis using the Japanese orthopaedic association back pain evaluation questionnaire. *Korean Journal of Spine*.

[7] Ungureanu G., Chitu A., Iancu I., Kakucs C., Maior T., Florian I. S. (2018). Gender differences in the self-assessment of quality of life and disability after spinal fusion for chronic low back pain at a neurosurgical center in Eastern Europe. *Neurospine*.

[8] Moon J. W., Shinn J. K., Ryu D., Oh S., Shim Y. S., Yoon S. H. (2017). Pelvic incidence can be changed not only by age and sex, but also by posture used during imaging. *Korean Journal of Spine*.

[9] Edwards R. D., Saladyga A. T., Schriver J. P., Davis K. G. (2012). Patient attitudes to surgeons' attire in an outpatient clinic setting: Substance over style. *The American Journal of Surgery*.

[10] Rehman S. U., Nietert P. J., Cope D. W., Kilpatrick A. O. (2005). What to wear today? Effect of doctor's attire on the trust and confidence of patients. *American Journal of Medicine*.

[11] Johnson S. C., Doi M. L. M., Yamamoto L. G. (2016). Adverse effects of tattoos and piercing on parent/patient confidence in health care providers. *Clinical Pediatrics*.

[12] Kanzler M. H., Gorsulowsky D. C. (2002). Patients' attitudes regarding physical characteristics of medical care providers in dermatologic practices. *JAMA Dermatology*.

[13] Mason L., Mason J. (2017). Patients' attitudes regarding characteristics of physicians in ophthalmology. *BMC Research Notes*.

[14] Abghari M. S., Takemoto R., Sadiq A., Karia R., Phillips D., Egol K. A. (2014). Patient perceptions and preferences when choosing an orthopaedic surgeon. *The Iowa Orthopaedic Journal*.

[15] Ardolino A., Williams L. A. P., Crook T. B., Taylor H. P. (2009). Bare below the elbows: what do patients think?. *Journal of Hospital Infection*.

[16] Lill M. M., Wilkinson T. J. (2005). Judging a book by its cover: Descriptive survey of patients' preferences for doctors' appearance and mode of address. *British Medical Journal*.

[17] Magos A., Maclean A., Baker D., Goddard N., Ogunbiyi O. (2007). Bare below the elbows: a cheap soundbite. *BMJ: British Medical Journal*.

[18] Yonekura C. L., Certain L., Karen S. K. K. (2013). Perceptions of patients, physicians, and Medical students on physicians' appearance. *Revista da Associação Médica Brasileira*.

[19] Yamada Y., Takahashi O., Ohde S., Deshpande G. A., Fukui T. (2010). Patients' preferences for doctors' attire in Japan. *Internal Medicine*.

[20] Kazory A. (2008). Physicians, their appearance, and the white coat. *American Journal of Medicine*.

[21] Loveday H. P., Wilson J. A., Pratt R. J. (2014). Epic3: national evidence-based guidelines for preventing healthcare-associated infections in nhs hospitals in England. *Journal of Hospital Infection*.

